# Comment on “Midwives' Role in Providing Nutrition Advice during Pregnancy: Meeting the Challenges? A Qualitative Study”

**DOI:** 10.1155/2019/4307214

**Published:** 2019-04-01

**Authors:** Angeliki Antonakou

**Affiliations:** Department of Midwifery, Midwifery School, “Alexander” Technological Educational Institute of Thessaloniki, Greece

Arrish et al. [1] reported some very interesting results in their qualitative study which are highly relevant to midwifery services throughout other countries in the world. It is true that midwives, despite their struggle and sincere intention to provide the best possible nutrition advice to women during pregnancy, do not manage to completely meet all the challenges. Arrish et al. reported that the role of midwives was felt to be constrained by many factors mostly out of the midwives' control. They suggested changes in the policy of maternity services such as allowing more time for antenatal visits, creating permanent positions for dietitians in antenatal clinics, and developing free online nutrition models and training packages for practising midwives by the professional organisations.

What was equally interesting in this study, however, was the fact that there were fifty-two midwives who initially expressed their interest in participating in this study, but in the end only sixteen were finally recruited (62.9% nonparticipation). This high drop-out rate might potentially reflect a lack of interest in the specific theme of this study in the younger age group of midwives as all but one final participant were over 35 years of age. It would be interesting to have known the demographics of this subgroup of midwives who declined to participate despite their initial interest so we could make further assumptions. Finally, in support of the findings of Arrish et al. we would like to add that the National Institute for Health and Care Excellence in 2015 issued a quality statement that lays the framework of nutritional advice provision in pregnancy [2]. It reports that all midwives should ensure that they give advice to pregnant women on how to eat healthily during pregnancy at their antenatal booking appointment. Moreover, pregnant women should receive this advice and support from a service that is evidence-based when informing them of the benefits of a healthy diet.
